# Genetic and Clinical Characterization of FLNC Variants in Chinese Patients with Cardiomyopathy

**DOI:** 10.3390/jcdd12120492

**Published:** 2025-12-12

**Authors:** Guofeng Xing, Li Chen, Lizhi Lv, Chengming Hu, Shengmei Liu, Yabing Duan, Jiachen Li, Qiang Wang, Xiaoyan Li

**Affiliations:** 1 Department of Pediatric Cardiac Surgery, Beijing Anzhen Hospital, Capital Medical University, No.2 Anzhen Street, Chaoyang District, Beijing 100029, China; xier2000@ccmu.edu.cn (G.X.); lvlihi@mail.ccmu.edu.cn (L.L.); happyhcm@mail.ccmu.edu.cn (C.H.); liushengmei1026@mail.ccmu.edu.cn (S.L.); helios_314029@mail.ccmu.edu.cn (Y.D.); lijiachen0915@mail.ccmu.edu.cn (J.L.); 2 Department of Pediatric Cardiology, Beijing Anzhen Hospital, Capital Medical University, No.2 Anzhen Street, Chaoyang District, Beijing 100029, China; liliforever@mail.ccmu.edu.cn; 3 Beijing Institution of Heart Lung and Blood Vassal Diseases, Beijing Anzhen Hospital, Capital Medical University, No.2 Anzhen Street, Chaoyang District, Beijing 100029, China

**Keywords:** cardiomyopathies, filamins, FLNC

## Abstract

This study investigates FLNC mutations in Chinese cardiomyopathy patients. Background: Inherited cardiomyopathies, including dilated cardiomyopathy (DCM), hypertrophic cardiomyopathy (HCM), restrictive cardiomyopathy (RCM), and arrhythmogenic right ventricular cardiomyopathy (ARVC) are major heart failure causes. FLNC, critical for muscle structure, is implicated in myofibrillar myopathy and isolated DCM (3–4% cases) with ventricular arrhythmias. Missense variants are linked to HCM and protein aggregation. A cohort of 25 patients with pathogenic/likely pathogenic FLNC mutations (2022–2025, Beijing Anzhen Hospital) underwent whole-exome sequencing (WES) using IDT kit 1.0/Hiseq 4000. Variants were classified via the American College of Medical Genetics and Genomics (ACMG) guidelines. Clinical data (echocardiography, CMR, labs) and follow-up data (prognosis, meds, and family history) were collected. The statistics used SPSS (*p* < 0.05). The mean age was 38 ± 14.6 years (13 males). There were 25 FLNC mutations: 12 single nucleotide polymorphisms (SNPs), 5 deletions, 2 duplications, and 3 deletion-insertions, classified as 6 pathogenic, 16 likely pathogenic, and 3 variants of uncertain significance (VUS). Diagnoses: 24% dilated cardiomyopathy (DCM), 8% hypertrophic cardiomyopathy (HCM), and 4% left ventricular non-compaction. Nonsense mutation carriers exhibited significantly higher tricuspid regurgitation prevalence compared to frameshift mutation carriers (6/9 vs. 2/10; *p* = 0.04). Echocardiography revealed reduced left ventricular ejection fraction (LVEF) (41.5 ± 14.1%), with statistically significant differences in fractional shortening (*p* = 0.024) and aortic root diameter (*p* = 0.028). Pedigree analysis confirmed that a frameshift mutation (LP) co-segregated with familial DCM and was associated with severe phenotypes, including sudden cardiac death. Furthermore, nonsense FLNC mutations correlated with increased tricuspid regurgitation severity, smaller aortic root dimensions, and reduced pulmonary artery flow velocity.

## 1. Introduction

Filamins, including FLNA, FLNB, and FLNC, are dimeric proteins consisting of 270–290 kDa subunits that crosslink filamentous (F-) actin [1,2]. Among these, FLNC stands out as one of the most important causes of inherited cardiomyopathies, which have emerged as a primary contributor to heart failure, significantly impacting morbidity, mortality, and the risk of sudden cardiac demise.

FLNC is uniquely and highly expressed in cardiac and skeletal muscle, featuring an Ig-like domain insertion that enables binding to sarcomeres at Z-discs. At these sites, FLNC crosslinks actin to anchor thin filaments, contributing to the structural integrity of muscle cells [3,4,5]. Since phosphorylation of FLNC by PKC diminishes calpain sensitivity, the assembly and disassembly of the Z-disc may be regulated by FLNC phosphorylation. Homozygous ablation of FLNC results in abnormal Z-disc morphology, compromised sarcomere integrity, and striated muscle dysfunction [6,7].

Over the past three decades, the identification of disease-inducing genetic mutations and advancements in unraveling the genetic underpinnings of cardiomyopathies have underscored their critical role in these devastating outcomes including FLNC [8]. Initially, FLNC was primarily associated with myofibrillar myopathy (MFM). However, subsequent high-throughput screening in cardiomyopathy cohorts revealed a prominent role for FLNC in isolated DCM [9].

Understanding the shared and distinct mechanisms of pathologic mutations in FLNC is a key step in investigating the mechanism of human heart failure. Given the significant impact of FLNC mutations on cardiac function, this study aimed to characterize the genetic and clinical features of FLNC-related cardiomyopathy in a cohort of Chinese patients.

## 2. Methods

### 2.1. Study Population

We performed whole exome sequencing (WES) of the proband presenting with heart failure and enrolled 25 cases with confirmed FLNC mutations. These patients were treated at Beijing Anzhen Hospital between January 2022 and January 2025. Clinical diagnoses and treatment were made based on the 2023 ESC Guidelines for the management of cardiomyopathies and 2021 ESC Guidelines for the diagnosis and treatment of acute and chronic heart failure [10,11]. The STROBE Checklist is in Appendix A Table A1.

### 2.2. Clinical Data Collection and Follow-Up

In this study, echocardiography results, cardiac magnetic resonance imaging (CMR) results, and laboratory test results of CK, CK-MB, hsTnI, CREA, BNP, and NT-proBNP were collected for each patient through their medical or hospitalization records at our center. Furthermore, follow-up interviews were conducted via telephone to gather information on patients’ prognoses, medication, and family genetic histories.

### 2.3. Whole-Exome Sequencing and Bioinformatic Analysis

Peripheral blood samples from patients and family members were collected. Genomic DNA was extracted using a DNA Blood Kit. Whole-exome sequencing (WES) was performed on the proband’s DNA with the IDT xGen Exome Research Panel v1.0 and sequenced on an Illumina HiSeq 4000 (Illumina, Inc., San Diego, CA, USA). Target regions achieved a mean coverage of ≥100×, with >99% of bases covered >20×.

Data were aligned to hg19 (NCBI build 37.1) using list BWA-MEM (v0.7.17). Variants were called via GATK (v4.6.2.0) and annotated with ANNOVAR (v2020-06-08) and SnpEff (v5.4.0a). Filters excluded variants with <10× coverage, heterozygous ratio < 35%, or base quality ≤ Q20.

Putative pathogenic FLNC variants (missense, nonsense, frameshift, splice-site) were prioritized. Rare variants (MAF < 0.01) were filtered against 1000 Genomes, ESP, and ExAC databases. Predictive tools (PolyPhen-2 (v2.2.3), SIFT (v6.2.1), PROVEAN (v1.1.5), MUpro (v1.0)) assessed pathogenicity, while GERP++ evaluated evolutionary conservation. Variants were classified per ACMG guidelines.

This workflow ensured robust identification of clinically relevant FLNC variants, balancing depth of characterization with computational rigor, and aligned with standards for rare genetic disorder research. The approach provided critical genotype-phenotype insights despite cohort size constraints.

### 2.4. Institutional Review Board Statement and Informed Consent Statement

This study followed the Helsinki Declaration, approved by Beijing Anzhen Hospital Ethics Committee (ZD2024010, 29 September 2024). IRB waived consent for this retrospective analysis of routine clinical data. No diagnostic/therapeutic changes or extra participant interactions occurred beyond standard care.

## 3. Statistical Analysis

Continuous variables are expressed as the mean values, and categorical variables are depicted using frequency. The difference between continuous variables was compared using the t-test. Comparisons of categorical variables between different groups were performed using the χ^2^ test or Fisher’s exact test. All statistical analyses in this study were performed using IBM SPSS Statistics 29.0.1.0 (IBM Corp., Armonk, NY, USA). A two-sided *p* value < 0.05 indicated statistical significance.

## 4. Results

### 4.1. Baseline Characteristics

The final analysis included 25 cases in the cohort. The average age of the patients is 38 years old, with the youngest being 9 years old and the oldest 65 years old. There are 13 male patients and 12 female patients. All 25 patients exhibited varying degrees of left ventricular enlargement and reduced left ventricular function and were diagnosed with heart failure.

All patients are taking pharmaceutical treatment including beta-blockers, empagliflozin or dapagliflozin, vericiguat, angiotensin II receptor antagonists, diuretics, and finerenone. 11 of 25 patients were successfully followed up. No patient underwent any surgical treatment, and only 2 patients had a family history of heart failure or DCM.

### 4.2. Whole Exome Sequence Analysis

Exome sequencing was performed on blood samples obtained from clinically suspected patients to analyze the FLNC gene. We identified 25 patients carrying FLNC mutations.

**Figure 1:**
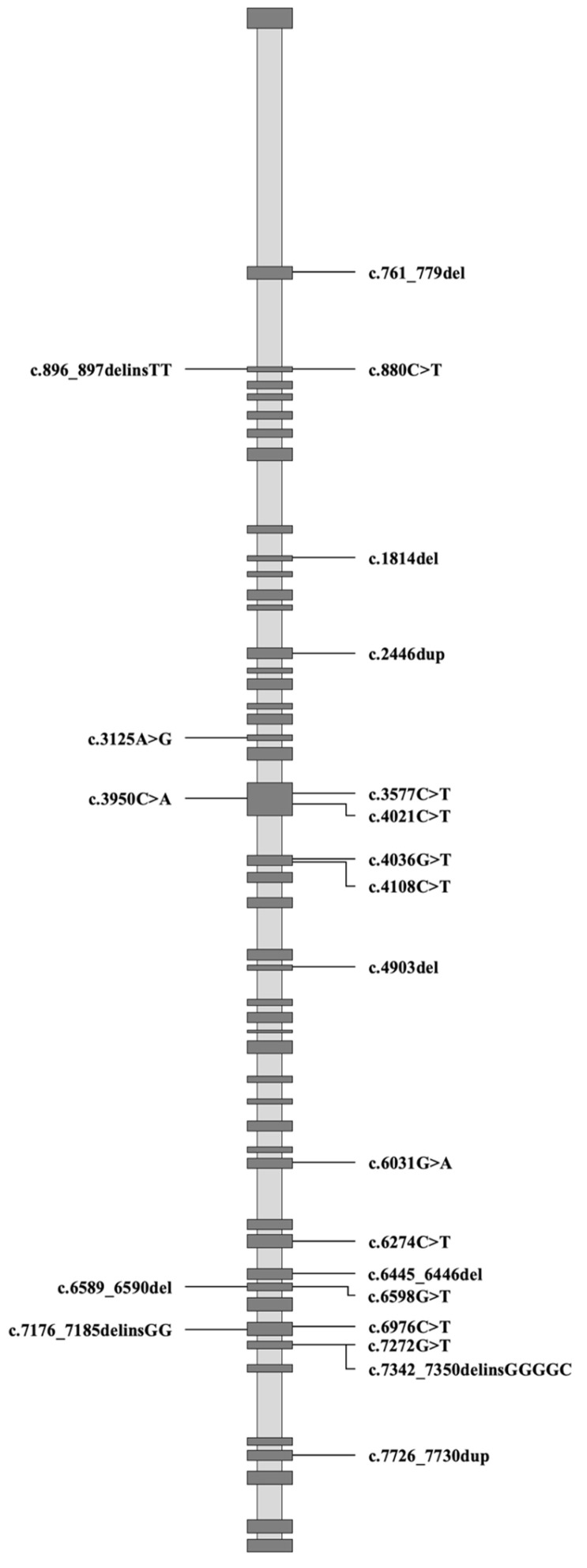
Mapping of Filamin C Variants.

The molecular consequences of these mutations included 9 nonsense mutations, 10 frameshift mutations, 4 missense mutations, and 2 splice site mutations. Notably, patient F19 carried 2 mutations of FLNC at the same time (c.6445_6446del and c.851-5C>A). 15 out of the 25 mutations had not been previously reported in the ClinVar database. All mutations were classified according to the ACMG Standards and Guidelines for Sequence Variant Interpretation, yielding the following categorization: 6 pathogenic variants (P), 16 likely pathogenic variants (LP), and 3 variants of uncertain significance (VUS). The detailed information regarding each mutation is presented in Table 1 and Figure 1.

### 4.3. Clinical Features

Among the 25 patients, 6 were diagnosed with dilated cardiomyopathy (DCM) (2 cases with frameshift mutations, 3 cases with nonsense mutations, and 1 with missense mutations), 2 with hypertrophic cardiomyopathy (HCM) (1 case with a frameshift mutation and 1 case with a missense mutation), and 1 with left ventricular non-compaction (LVNC) (carrying a missense mutation). Furthermore, 5 patients had varying degrees of left and/or right ventricular wall fibrosis. 7 patients had concurrent type 2 diabetes mellitus, 2 had hypercholesterolemia, 3 had cardiac arrhythmia, and 1 had coronary atherosclerotic heart disease. Additionally, out of the 7 diabetic patients, 3 were suffering from stage I diabetic nephropathy.

Eighteen patients exhibited mild to moderate mitral and/or tricuspid regurgitation. Among the 9 patients with nonsense variants, 6 demonstrated tricuspid valve involvement, whereas only 2 out of 10 patients with frameshift variants showed similar involvement. In our exploratory analysis, we observed a trend towards a higher prevalence of tricuspid regurgitation in patients carrying nonsense variants compared to those with frameshift variants (6/9 vs. 2/10; *p* = 0.040). However, given the small number of events, this finding must be interpreted with caution and warrants further investigation.

According to the echocardiogram result, the average left ventricular ejection fraction (LVEF) among the 25 cases was 41.5%, 35.6% in the nonsense variants group, and 43.4% in the other variants group (*p* = 0.229). 9 cases have an LVEF lower than 35.0%, 5 cases belong to the nonsense group, and 4 cases belong to other variants group. The average left ventricular end-diastolic diameter (LVEDD) was 67.3 mm, 62.8 mm in the nonsense group, and 69.9 mm in the other variants group (*p* = 0.615). The average left ventricular end-systolic diameter (LVESD) was 52.6 mm, 50.0 mm in the nonsense group, and 54.2 mm in the other variants group (*p* = 0.727). The average fraction of shortening (FS) was 22.6 ± 9.5%, with a maximum of 39.1% and minimum of 10.7%. The average of FS is 21.1% in the nonsense group and 23.5% in the frameshift group (*p* = 0.568). The diameter of the root of the aorta is 30.0 mm, 27.8 mm in the nonsense group, and 31.5 mm in the frameshift group (*p* = 0.041). The pulmonary artery flow velocity is 90.1 cm/s, 79.1 cm/s in the nonsense group, and 97.1 cm/s in the frameshift group (*p* = 0.010). (Table 2)

### 4.4. Pedigree Analysis of Patient F20

**Figure 2:**
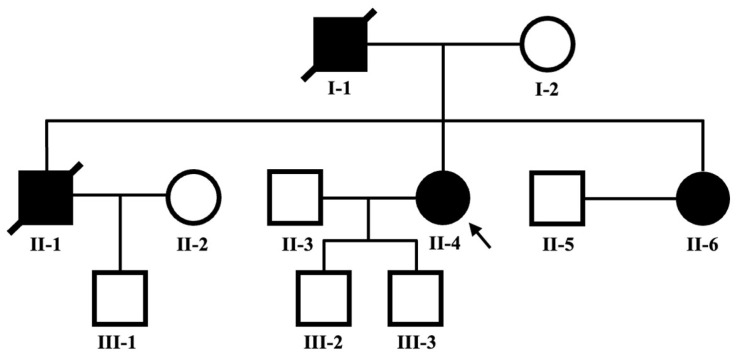
Pedigree Chart of Patient F20 (II4).

**Figure 3:**
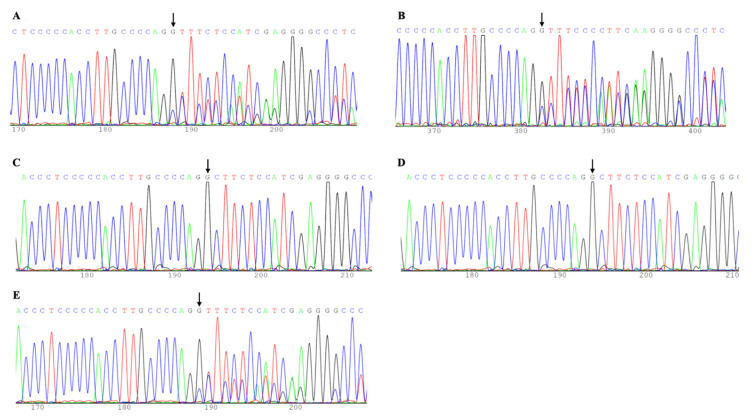
Sequencing Chromatograms for the FLNC Gene c.1814del Variant. (**A**): II4, (**B**): II6, (**C**): III1, (**D**): III2, and (**E**): II6.

We also performed a pedigree analysis of FLNC-related familial DCM on patient F20 (II4), with the pedigree data illustrated in Figure 2. Sanger sequencing confirmed that family members II4 (proband), II6 (proband’s younger sister), and III3 (proband’s son) carry the same frameshift mutation (Figure 3), with the variant showing co-segregation with the clinical phenotype within the pedigree. According to ACMG guidelines, this variant is classified as a LP variant (PVS1 + PM2).

In this three-generation family, all affected members, including I-1, II-1, II-4, and II-6 exhibit DCM with reduced Left Ventricular Ejection Fraction (LVEF) ranging from 20% to 65% and increased Left Ventricular End-Diastolic Diameter (LVEDd up to 54 mm). Ventricular septal thickness is elevated in generation III (11.0–11.2 mm). Electrophysiological abnormalities including T-wave flattening (II-4), non-specific intraventricular conduction delay (II-6), polymorphic ventricular premature beats (II-4, II-6), and sinus bradycardia (III-4) are present. It is also notable that individual II-1 experienced sudden cardiac death.

## 5. Discussion

The expanding genetic and clinical spectrum of FLNC-related cardiomyopathy underscores its complex phenotypic heterogeneity and therapeutic challenges [12]. In this study, we documented genetic data and clinical records from 25 heart failure patients with FLNC mutations to investigate phenotypic disparities linked to distinct mutation types.

Notably, our cohort revealed numerous FLNC variants absent from the ClinVar database (Section 4.2 Results and Table 1). This high proportion of novel variants suggests that the genetic architecture of FLNC cardiomyopathy in the Chinese population may be broader and more diverse than previously described in predominantly Caucasian cohorts. These findings underscore the importance of population-specific genetic research to elucidate unique mutational patterns and phenotypic associations, ultimately advancing precision medicine strategies for this rare cardiac disorder.

Within the FLNC protein structure, the ROD1 (R3–R5, 469–759) and ROD2 (R16–R21, 1759–2403) regions play distinct functional roles, and mutations in these domains are associated with variable cardiomyopathies [12,13]. The ROD2 domain is implicated in cell signaling, and missense variants here correlate with HCM. And variants in the ROD1 domain are linked to DCM, RCM, etc. [14]. In our cohort of 21 patients with FLNC mutations, 13 cases (52.0%) exhibited mutations affecting the ROD2 domain, 2 cases (8.0%) demonstrated combined mutations impacting both ROD1 and ROD2 domains, 1 case (4.0%) exhibited mutations affecting the ROD1 domain, and 9 cases (36.0%) carried mutations located outside these critical structural regions (affecting neither ROD1 nor ROD2). This observation suggests that ROD2 domain mutations may play a more dominant role in the pathogenesis of FLNC-associated cardiomyopathies, as they constituted the majority (83.3%) of P and LP variants identified in our cohort.

Research has demonstrated that distinct types of FLNC gene variants exhibit varying clinical characteristics. Truncating variants of FLNC are found in approximately 3 to 4% of patients with DCM which presents in early-to-mid adulthood and is associated with a high rate of ventricular arrhythmias and sudden cardiac death [15,16,17]. Missense variants are more commonly associated with HCM and can cause pathologic FLNC protein aggregation [14]. In our cases, the family of patient F20 has shown a predisposition toward DCM, arrhythmic risk, and cardiac sudden death. Other patients with nonsense mutations exhibited a statistically significant lower pulmonary artery flow velocity (79.1 cm/s vs. 97.1 cm/s, *p* = 0.010), a smaller aortic root diameter (27.8 mm vs. 31.5 mm, *p* = 0.041) compared to carriers of frameshift mutations, and a higher prevalence of tricuspid regurgitation (*p* = 0.040). Studies have indicated that filamin proteins (FLNs) contribute to aortic wall structural integrity and related to valvular disorder [18,19,20].

We propose that FLNC may potentially modulate aortic root and valvular architecture via analogous mechanisms, though the precise molecular pathways underlying this diameter discrepancy require further elucidation. In our Chinese cohort, FLNC nonsense mutations appear to be associated with a distinctive right-heart phenotypic profile—characterized by notable tricuspid regurgitation and diminished pulmonary artery flow velocity—which may extend the conventional understanding of FLNC truncating variants that have traditionally centered on arrhythmia and left ventricular dilation. While pulmonary artery diameter demonstrated no statistically significant differences, the observed reduction in pulmonary flow velocity could suggest a preferential association between nonsense mutations and right ventricular dysfunction. These preliminary observations, coupled with the high frequency of novel variants in our cohort, highlight the importance of cautious interpretation and the need for larger, population-specific studies to better delineate genotype-phenotype relationships and their implications for clinical management in FLNC-related cardiomyopathy. Further functional investigations and cross-cohort comparisons would be valuable to confirm these putative associations and explore their mechanistic underpinnings [16].

## 6. Conclusions

Our study investigates FLNC-related cardiomyopathies, revealing phenotypic diversity linked to mutation location and type. Nonsense mutations show higher prevalence of tricuspid regurgitation, smaller aortic roots, and lower pulmonary artery flow velocity compared to other mutations, suggesting truncating variants correlate with impaired ventricular systolic function and FLNC may influence aortic integrity.

## Figures and Tables

**Table 1:** Details of Mutations in the Cohort.

PatientID	Gene	Mutations		Pathogenicity	ACMG
F01	FLNC	c.6445_6446del	p.R2197Gfs*47	LP	PVS1 PM2
F02	FLNC	c.4021C>T	p.Arg1341X	P	PVS1 PS4 PM2
F03	FLNC	c.4108C>T	p.R1370X	P	PVS1 PS4 PM2
F04	FLNC	c.761_779del	p.Leu254Profs*14	LP	PVS1 PM2
F05	FLNC	c.5199+1G>C		P	PVS1 PS4 PM2
F06	FLNC	c.4108C>T	p.R1370X	P	PVS1 PS4 PM2
F07	FLNC	c.4036G>T	p.E1346X	LP	PVS1 PM2
F08	FLNC	c.880C>T	p.Q294X	P	PVS1 PS4 PM2
F09	FLNC	c.7342_7350delinsGGGGC	p.S2448Gfs*	LP	PVS1 PM2
F10	FLNC	c.3577C>T	p.A1186V	LP	PS2 PM2
F11	FLNC	c.2446dup	p.D816fs	LP	PVS1 PM2
F12	FLNC	c.6031G>A	p.G2011R	LP	PS2 PM2 PP3
F13	FLNC	c.6274C>T	p.Q2143X	LP	PVS1 PM2
F14	FLNC	c.6976C>T	p.R2326X	P	PVS1 PS4 PM2
F15	FLNC	c.4903del	p.D1635fs	LP	PVS1 PM2
F16	FLNC	c.7726_7730dup	p.Y2577fs	LP	PVS1 PM2
F17	FLNC	c.6598G>T	p.E2200X	LP	PVS1 PM2
F18	FLNC	c.7272G>T	p.E2458X	LP	PVS1 PM2
F19	FLNC	c.6445_6446del	p.T2149fs	LP	PVS1 PM2
		c.851-5C>A		VUS	PM2 PP3
F20	FLNC	c.1814del		LP	PVS1 PM2
F21	FLNC	c.7176_7185delinsGG	p.IIe2392Metfs*13	LP	PVS1 PM2
F22	FLNC	c.1211-1G>A		LP	PVS1 PM2
F23	FLNC	c.896_897delinsTT	p.Thr299Ile	VUS	PM2
F24	FLNC	c.3950C>A	p.Thr1317Asn	VUS	PM2 PP3
F25	FLNC	c.3125A>G	p.Tyr1042Cys	VUS	PM2 PP3

The table lists the specific FLNC mutations found in the 25 patients, including the genomic (HGVS c.) and protein (HGVS p.) nomenclature, their pathogenicity classification, and the supporting American College of Medical Genetics and Genomics (ACMG) criteria. Abbreviations: LP, Likely Pathogenic; P, Pathogenic; VUS, Variant of Uncertain Significance; PVS1, null variant criteria; PS4, phenotype specificity criteria; PM2, absent from controls criteria; PP3, computational evidence criteria. * signifies a premature stop codon.

**Table 2:** Baseline and Clinical Features of the Cohort.

	Total Population (*n* = 25)	Nonsense Variants (*n* = 9)	Other Variants (*n* = 16)	*p* Value
Age, y	38.0 (39.0)	38.5 (36.0)	37.4 (40.0)	
Male	13	4	9	
LVEF, %	41.5 (38.5)	35.6 (30.0)	43.4 (40.0)	0.229
LVEF ≤ 35%	9	5	4	0.127
FS, %	22.6 (20.1)	21.1 (13.9)	23.5 (20.4)	0.568
LVEDd, mm	67.3 (61.0)	62.8 (65.0)	69.9 (60.0)	0.615
LVESd, mm	52.6 (48.5)	50.0 (54.0)	54.2 (47.0)	0.727
Ventricular Spectrum, mm	7.8 (8.0)	7.3 (7.6)	8.2 (8.0)	0.216
Aortic Root, mm	30.0 (31.0)	27.8 (31.0)	31.5 (31.5)	0.041
Aortic Flow Velocity, cm/s	129.9 (133.0)	120.7 (123.0)	135.8 (138.0)	0.191
Pulmonary Artery, mm	22.9 (23.0)	23.1 (21.0)	22.7 (24.0)	0.767
Pulmonary Artery Flow Velocity, cm/s	90.1 (90.0)	79.1 (77.0)	97.1 (92.5)	0.010

Continuous variables are presented as mean (median); categorical variables are presented as counts. Group comparisons were performed using the independent samples *t*-test for continuous variables and the χ^2^ test or Fisher’s exact test for categorical variables. A two-sided *p* value < 0.05 was considered statistically significant. Abbreviations: LVEF, Left Ventricular Ejection Fraction; FS, Fractional Shortening; LVEDd, Left Ventricular End-Diastolic Diameter; LVESd, Left Ventricular End-Systolic Diameter.

## Data Availability

The original contributions presented in this study are included in the article. Further inquiries can be directed to the corresponding author(s).

## Appendix A

**Table A1:** The STROBE Checklist of Cross-Sectional Studies.

	Item No.	Recommendation	Page No.
**Title and abstract**	1	(a) Indicate the study’s design with a commonly used term in the title or the abstract	1
		(b) Provide in the abstract an informative and balanced summary of what was performed and what was found	1
**Introduction**			
Background/rationale	2	Explain the scientific background and rationale for the investigation being reported	1–2
Objectives	3	State specific objectives, including any prespecified hypotheses	1–2
**Methods**			
Study design	4	Present key elements of study design early in the paper	2
Setting	5	Describe the setting, locations, and relevant dates, including periods of recruitment, exposure, follow-up, and data collection	2
Participants	6	(a) Give the eligibility criteria, and the sources and methods of selection of participants	2
Variables	7	Clearly define all outcomes, exposures, predictors, potential confounders, and effect modifiers. Give diagnostic criteria, if applicable	2
Data sources/measurement	8	For each variable of interest, give sources of data and details of methods of assessment (measurement). Describe comparability of assessment methods if there is more than one group	*2*
Bias	9	Describe any efforts to address potential sources of bias	2
Study size	10	Explain how the study size was arrived at	2
Quantitative variables	11	Explain how quantitative variables were handled in the analyses. If applicable, describe which groupings were chosen and why	2–3
Statistical methods	12	(a) Describe all statistical methods, including those used to control for confounding	2
		(b) Describe any methods used to examine subgroups and interactions	2
		(c) Explain how missing data were addressed	2
		(d) If applicable, describe analytical methods taking account of sampling strategy	Not applicable
		(e) Describe any sensitivity analyses	
**Results**			
Participants	13	(a) Report numbers of individuals at each stage of study—e.g., numbers potentially eligible, examined for eligibility, confirmed eligible, included in the study, completing follow-up, and analyzed	2
		(b) Give reasons for non-participation at each stage	
		(c) Consider use of a flow diagram	
Descriptive data	14	(a) Give characteristics of study participants (e.g., demographic, clinical, social) and information on exposures and potential confounders	2
		(b) Indicate number of participants with missing data for each variable of interest	
Outcome data	15	Report numbers of outcome events or summary measures	2
Main results	16	(a) Give unadjusted estimates and, if applicable, confounder-adjusted estimates and their precision (e.g., 95% confidence interval). Make clear which confounders were adjusted for and why they were included	2
		(b) Report category boundaries when continuous variables were categorized	2
		(c) If relevant, consider translating estimates of relative risk into absolute risk for a meaningful time period	2
Other analyses	17	Report other analyses performed—e.g., analyses of subgroups and interactions, and sensitivity analyses	3–7
**Discussion**			
Key results	18	Summarize key results with reference to study objectives	7–8
Limitations	19	Discuss limitations of the study, taking into account sources of potential bias or imprecision. Discuss both direction and magnitude of any potential bias	7–8
Interpretation	20	Give a cautious overall interpretation of results considering objectives, limitations, multiplicity of analyses, results from similar studies, and other relevant evidence	7–8
Generalisability	21	Discuss the generalisability (external validity) of the study results	7–8
**Other information**			
Funding	22	Give the source of funding and the role of the funders for the present study and, if applicable, for the original study on which the present article is based	8
